# Overuse-related instability of the elbow: the role of CT-arthrography

**DOI:** 10.1186/s13244-021-01065-8

**Published:** 2021-10-11

**Authors:** Andrea Zagarella, Giulia Signorelli, Giulia Muscogiuri, Roberta Colombo, Gianluca Folco, Paolo Arrigoni, Mattia Radici, Pietro Simone Randelli, Mauro Battista Gallazzi

**Affiliations:** 1 U.O.C. Radiodiagnostica, Azienda Socio Sanitaria Territoriale Centro Specialistico Ortopedico Traumatologico Gaetano Pini-CTO, Piazza Cardinal Ferrari 1, 20122 Milan, Italy; 2 Scuola Di Specializzazione in Radiodiagnostica, Università Degli Studi Di Milano, Via Festa del Perdono 7, 20122 Milan, Italy; 3 I Clinica Ortopedica, Azienda Socio Sanitaria Territoriale Centro Specialistico Ortopedico Traumatologico Gaetano Pini-CTO, Piazza Cardinal Ferrari 1, 20122 Milan, Italy; 4 Scuola Di Specializzazione in Ortopedia e Traumatologia Università Degli Studi Di Milano, Via Festa del Perdono 7, 20122 Milan, Italy; 5 Laboratory of Applied Biomechanics, Department of Biomedical Sciences for Health, Università Degli Studi Di Milano, Via Mangiagalli 31, 20133 Milan, Italy; 6 U.O.C. 1° Clinica Ortopedica, ASST Centro Specialistico Ortopedico Traumatologico Gaetano Pini-CTO, Piazza Cardinal Ferrari 1, 20122 Milan, Italy; 7 Research Center for Adult and Pediatric Rheumatic Diseases (RECAP-RD), Department of Biomedical Sciences for Health, Università Degli Studi Di Milano, Via Mangiagalli 31, 20133 Milan, Italy

**Keywords:** Elbow anatomy, Elbow instability, CT-arthrography, Ligament injury, US-guided injection

## Abstract

The elbow is a complex joint whose biomechanical function is granted by the interplay and synergy of various anatomical structures. Articular stability is achieved by both static and dynamic constraints, which consist of osseous as well as soft-tissue components. Injuries determining instability frequently involve several of these structures. Therefore, accurate knowledge of regional anatomy and imaging findings is fundamental for a precise diagnosis and an appropriate clinical management of elbow instability. This review focuses particularly on the varied appearance of overuse-related elbow injuries at CT-arthrography.

## Keypoints

- Overuse-related elbow injuries are commonly encountered in both athletes and nonathletes.
- An accurate radiological diagnosis contributes to the appropriate management of elbow instability.
- Multimodality imaging is recommended when studying elbow instability.
- CT-arthrography represents a solid choice whenever MRI is unavailable or clinically unfeasible.
- Supplementary dynamic US evaluation of ligaments through stress testing is diagnostically relevant.

## Background

Even though magnetic resonance imaging (MRI) is considered to be the gold standard for the detection of ligament injuries of the elbow, computed tomography (CT) imaging is usually more readily available and can be alternatively used, in certain clinical settings, for a proper diagnosis of elbow instability.

In this imaging review of elbow instability patterns, we will first discuss critical aspects of elbow anatomy; then, we will describe the technique of ultrasound (US)-guided CT-arthrography, with the scope of providing a useful reference for radiologists not accustomed to this particular imaging modality. Lastly, we will provide a series of imaging findings pertaining to the most common scenarios of overuse-related instability.

## Anatomy

The elbow consists of three main joints enclosed in a single synovial capsule, providing two degrees of freedom of motion: the humero-ulnar, the humero-radial, and the radio-ulnar joints [1].

The main determinant of elbow stability is the humero-ulnar joint, with the coronoid process of the ulna playing an important role.

The fulcrum of flexion and extension movements is the humero-ulnar joint, whereas pronation and supination have their fulcrum at the radio-ulnar joint. The physiological range of motion of these movements is up to 140° for flexion–extension and up to 180° for supination-pronation [2, 3].

**Fig. 1:**
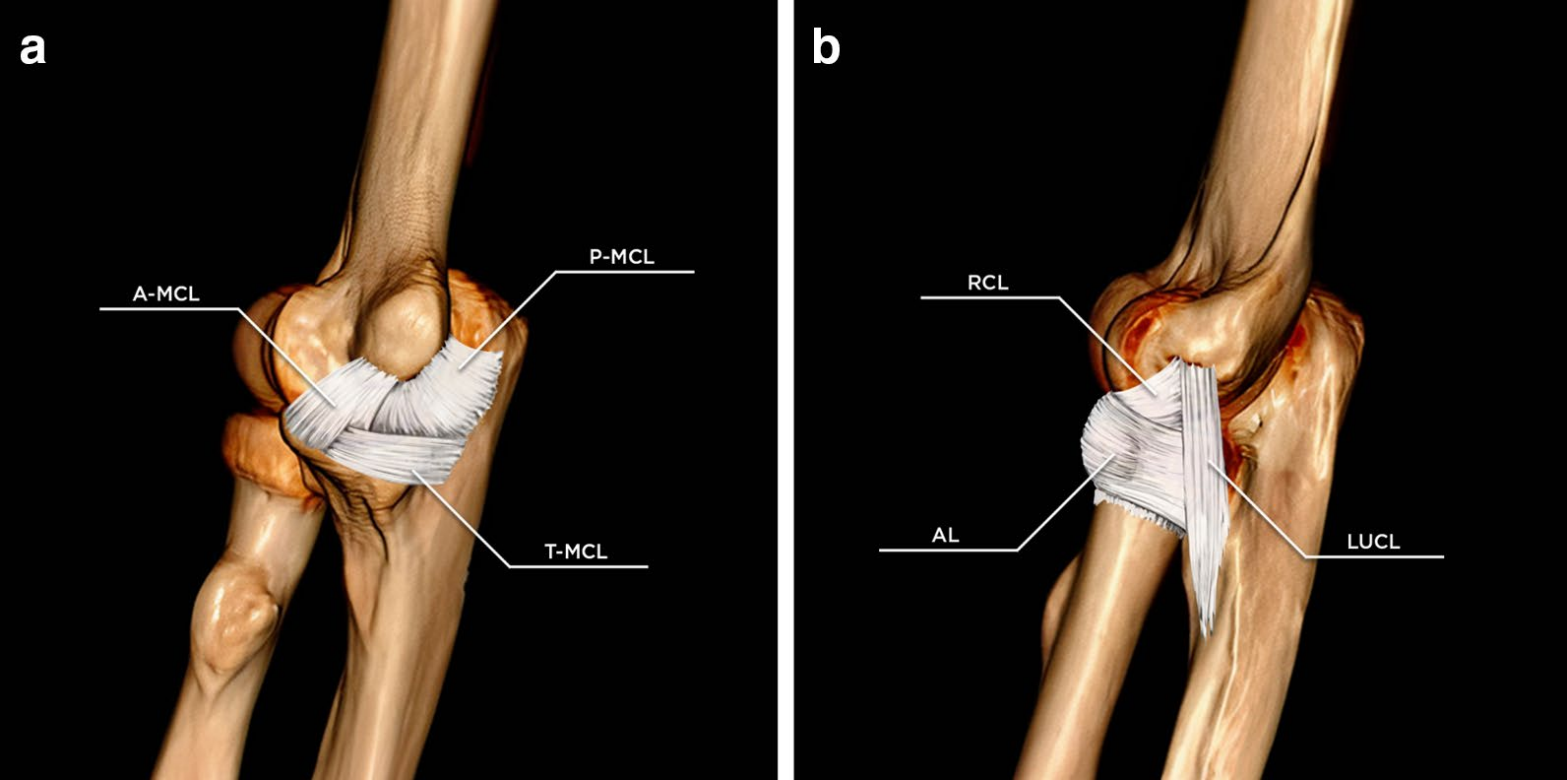
Elbow 3D reformatting with ligament illustrations. Medial view (**a**). The medial collateral ligament (MCL) complex and its components: the anterior bundle of the medial collateral ligament (A-MCL), the posterior bundle of the medial collateral ligament (P-MCL), and the transverse ligament (T-MCL). Lateral view (**b**). The lateral collateral ligament (LCL) complex and its components: the radial collateral ligament (RCL), the lateral ulnar collateral ligament (LUCL), and the annular ligament (AL)

The ligamentous supporting structures of the elbow originate as focal thickenings of the articular capsule and are divided into medial and lateral complexes, which provide stability under valgus and varus stress, respectively [4, 5]. The medial or ulnar collateral ligament complex (MCL) is constituted by an anterior, a posterior, and a transverse bundle (Fig. 1a). In 23% of individuals, an accessory ulnar collateral ligament can be found: it arises from the posterior articular capsule, inserting onto the transverse bundle [6, 7].

The anterior bundle (A-MCL) originates from the lower border of the medial epicondyle, inserting at the sublime tubercle of the ulnar coronoid process; it is constituted by a superficial and a deep layer. The former, however, is a different structure from the articular capsule, as it is associated with deep fibers of the flexor digitorum superficialis tendon [8]. The anterior bundle can also be separated into two bands, characterized by different degrees of tautness across the flexion/extension range of motion [9]; the A-MCL is the main soft tissue stabilizer against valgus forces applied to the elbow [10, 11], particularly between 20° and 120° flexion angles [12–14]. On the other hand, at low degrees of flexion (< 20°), the main contribution to stability is granted by bony congruity: anteriorly, the ulnar coronoid process articulates with the humeral coronoid fossa, whereas posteriorly, the olecranon articulates with the olecranon fossa [12, 13, 15, 16]. The posterior bundle (P-MCL) arises posteriorly from the humeral medial epicondyle, attaching medially to the olecranon, where it forms the floor of the cubital tunnel. The P-MCL is the main determinant of elbow stability at flexion angles above 120° [12, 13]. The transverse bundle (T-MCL) arises from the medial aspect of the proximal olecranon and inserts distally to the coronoid. The contribution of the T-MCL to stability is minimal, as it both originates and inserts on the ulna [7, 16]. Moreover, the T-MCL is characterized by anatomical variability and is not always present, making it relatively unimportant from a clinical-radiological standpoint.

On the lateral side of the elbow joint, the radial or lateral collateral ligament (LCL) complex (Fig. 1b) is the main stabilizer against varus and external rotational forces. The LCL, presenting with some interindividual variability [17–19], is a Y-shaped structure constituted by the radial collateral ligament (RCL), the annular ligament (AL), and the lateral ulnar collateral ligament (LUCL). In around 30% of individuals, an accessory lateral collateral ligament can be seen, coursing from the annular ligament to the ulnar supinator crest [6, 7]. The RCL is a ligament with a fan-shaped morphology which arises from the lateral epicondyle; it courses longitudinally beneath the common extensor tendon and blends anteriorly with the annular ligament. The RCL is isometric in nature and best visualized on coronal scans [6]. The annular ligament (AL) both originates and inserts on the sigmoid notch of the ulna, encompassing the anterior aspect of the radial head; it is best visualized on axial and sagittal scans [6, 19]. The AL is crucial for stabilization of the radial head together with the ulna, throughout the pronation-supination range of motion of the forearm; its morphology can vary, as it is generally not constituted by a uniform band. The LUCL arises from the lateral epicondyle, partially blending in with the annular ligament as it courses distally towards its insertion on the ulnar supinator crest [6, 7, 19]; it is best visualized on coronal and sagittal scans [6]. The LUCL, taut throughout the flexion–extension arc, used to be regarded as the main lateral stabilizer of the elbow [18]. Recent biomechanical studies have shown instead that the LCL complex as a whole contributes substantially towards stability of the elbow, by virtue of its interconnected Y-shaped morphology, hinting at the fact that an isolated lesion of the LUCL is not sufficient to result in postero-lateral rotatory instability of the elbow (PLRI) [19–24]. Nevertheless, it is undeniable that the aforementioned ligaments, aided by static and dynamic support provided by the extensor muscles, constitute the primary elbow stabilizer against rotatory instability.

Osseous stabilizers are also worth mentioning, as the radial head is instrumental in preventing external subluxation of the elbow, by providing tension to the LCL complex. Studies have shown that isolated resection of the radial head leads to increased external rotator patholaxity, even if the ligaments remain intact [25–27].

## The role of CT-arthrography

CT provides important information in the evaluation of the musculoskeletal system; nevertheless, the main role of musculoskeletal CT is usually related to the study of bone, while soft tissue injuries are routinely studied via US or MRI. In particular, MRI is a well-established and efficacious imaging modality for the assessment of ligaments, as it ensures high-resolution soft tissue contrast while also allowing simultaneous evaluation of bone anatomy [28].

MRI is therefore considered a first-choice imaging technique for the evaluation of the musculoskeletal system, providing a panoramic overview of both intra- and extra-articular structures. Specific joints require injection of intra-articular contrast agent to obtain capsular distension (MR-arthrography). A proper tension on ligaments allows for better visualization, especially when combined with the high intensity signal of the contrast agent (gadolinium) on T1-weighted sequences, appearing as thin, hypointense bands, highlighted by a distended and contrast-filled articular cavity.

Similarly, CT-arthrography enables a better evaluation of intra-articular structures when compared to CT alone [29], granted by the elevated contrast between high-density iodinated medium and low-density anatomical structures, such as cartilage and ligaments.

Although plain MRI remains the reference standard for a wide range of musculoskeletal disorders, MR-arthrography may prove particularly useful in specific circumstances, e.g. laxity of the LCL or MCL.

Even though MR-arthrography is usually preferred to CT-arthrography due to its elevated soft-tissue contrast resolution, CT-arthrography provides better spatial resolution, lower acquisition times, and is the modality of choice for select patients [30]. In particular, CT-arthrography has been shown by Klaan et al. to have an excellent diagnostic accuracy for the detection of cartilage defects, osteochondral lesions, and intra-articular loose bodies, roughly equivalent to that of MR-arthrography [31]. Moreover, the higher spatial resolution of CT-arthrography, combined with the use of high-density iodinated contrast, enhances the visualization of intra-articular structures and increases diagnostic accuracy of small joint cartilage lesions (wrist, elbow, ankle); in this specific setting, CT-arthrography is considered superior to MR-arthrography [31, 32].

Excellent candidates for CT-arthrography include individuals carrying pacemakers or non-MRI safe implantable devices and patients affected by claustrophobic anxiety disorders or who cannot tolerate gadolinium-based contrast medium [33].

CT-arthrography is a high-quality imaging technique, especially with the supplemental use of multiplanar processed images, which allow for a better assessment of regional anatomy. In particular, the use of an isotropic voxel results in high-quality multiplanar reformatting without spatial distortion. Accurate ligament imaging can thus be obtained on every desired plane, regardless of their spatial orientation, by ad-hoc reformatting of the acquired volume. Even though isotropic voxel imaging can also be obtained using MRI, acquisition times are undoubtedly longer than CT. This may result problematic in some clinical instances (e.g. claustrophobia, severe pain); on the other hand, modern-day spiral CT scanners can acquire the desired anatomical volume in a matter of a few seconds, as opposed to a volumetric MRI acquisition that lasts several minutes.

Generally, detection of ligament injuries and diagnosis of elbow instability would be made through CT-arthrography in cases where MRI or MR-arthrography results had been equivocal. In practice, however, CT is often preferred to MRI due to its increased availability worldwide, which occurred over the last 20 years [34, 35], and due to the lower costs and shorter times involved. The advent of multi-detector helical scanners has resulted in lower scanning times and higher quality images: scans that previously required several minutes are now completed in mere seconds.

One of the major disadvantages of CT is obviously the exposure to ionizing radiation. Nevertheless, patients presenting with elbow disorders are subjected to a lower radiation exposure nowadays. In support of this, it is strongly recommended that radiologists perform elbow CT scans with the arm elevated overhead, when feasible. This results in a significantly lower dose of absorbed radiation compared to the arm adjacent to the torso; Iordache et al. analyzed elbow CT scans performed with an overhead arm, obtaining an effective dose of 0.158 mSv [36].

Thus, while MR-artrography remains the gold standard in the evaluation of elbow articular disorders, CT-arthrography can be considered a valid alternative imaging modality, especially in the case of newer generation multi-slice CT scanners and in patients with contraindications to MRI scanning [37].

Intra-articular contrast agent injection prior to CT-arthrography should always be performed with an imaging-guided technique rather than a palpation-guided technique, since it results in more accurate intra-articular delivery, as confirmed by Kim et al. [38].

Different imaging modalities can be used to perform the intra-articular injection; US guidance is now favored over fluoroscopy by many specialists [39].

US is an easy, cheap, effective, and radiation-free imaging modality that can be used to perform interventional procedures, such as intra-articular injections. US guidance of interventional musculoskeletal procedures has been shown to be safe and presents several advantages [40–42]. The needle can be maneuvered in real time, unlike with fluoroscopy guidance, where it can only be observed intermittently. Moreover, the tip can be clearly seen upon entering the joint space and the injection of iodinated contrast can be closely monitored, as it distends the articular recess.

Portable US systems may be placed directly in medication rooms adjacent to the CT room; a dedicated room is not strictly required for the US-guided procedure, reducing the delay between injection and CT-arthrography to a minimum. Furthermore, a pre-procedural US evaluation, combining static and dynamic scanning, is a valuable diagnostic tool complementary to CT-arthrography and is essential for radiologists who want to provide patients with an exhaustive, imaging-integrated evaluation of the elbow.

This review focuses on the US-guided CT-arthrography imaging appearance of overuse-related injuries of the elbow involving ligaments.

## Technique

As with any invasive procedure, informed written consent must be acquired prior to the injection.

Before intra-articular injection of the iodinated contrast agent, a preliminary US scan is performed for the evaluation of the articular recesses, ligaments and tendons. A linear multi-frequency (3–13 MHz) ultrasound transducer by Esaote (MyLab Class C scanner) was employed.

In order to perform the US-guided injection, the patient is positioned prone on the scanning table, with the affected limb flexed at 90° beyond the margin of the table.

A sterile technique is mandatory. Aseptic preparation of the skin is obtained in a sterile field, while employing strictly sterile disposable materials: gloves, probe cover, ultrasound gel, needles and syringes.

**Fig. 2:**
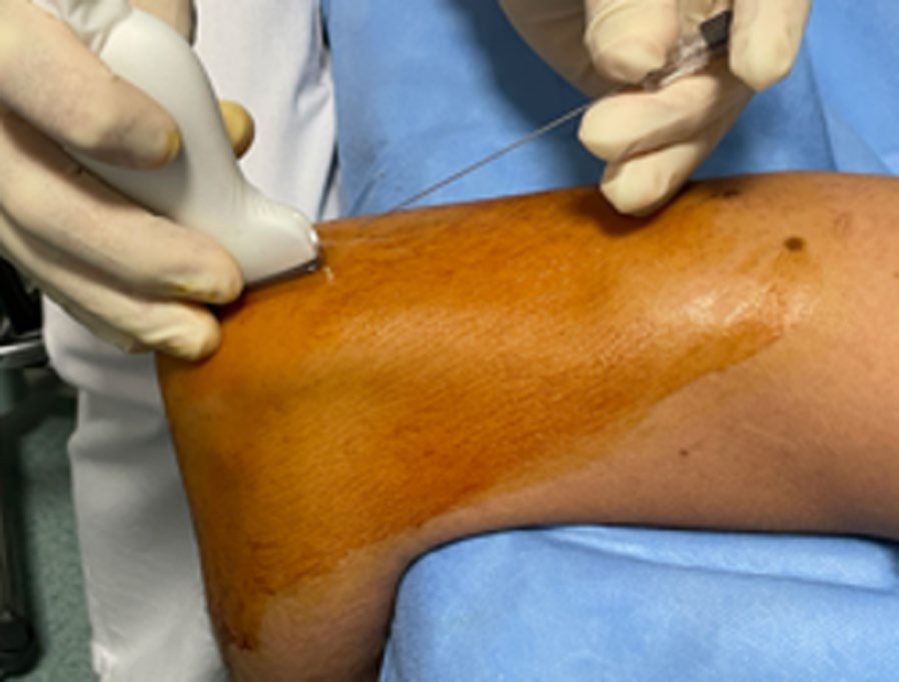
In-plane US-guided contrast agent injection using a posterior access. The spinal needle is guided through the triceps tendon, with an approximate 30° angle between the needle and the probe in order to obtain an optimal visualization of the needle tip advancing to the posterior articular recess

The injecting needle (22 G, 0.7 × 70 mm spinal needle) is positioned with an in-plane modality in order to follow its trajectory from the access point to the target site (Fig. 2).

The probe is placed longitudinally over the posterior region of the elbow, in order to visualize the olecranon fossa. The spinal needle is then positioned parallel to the midline of the long axis of the transducer. A trans-tricipital access is then performed, with an approximate 30° angle between the needle and the probe for an optimal visualization of the tip of the needle proceeding towards the posterior articular recess at the olecranon fossa.

The posterior access is recommended to avoid contrast agent extra-articular spillage around the radial collateral ligaments, which may impair a correct diagnosis; this circumstance may take place by injecting the contrast with a lateral approach through the radio-humeral articular space.

An anesthetic agent (2–3 mL of lidocaine hydrochloride) may be administered locally to reduce the discomfort of intra-articular puncture.

**Fig. 3:**
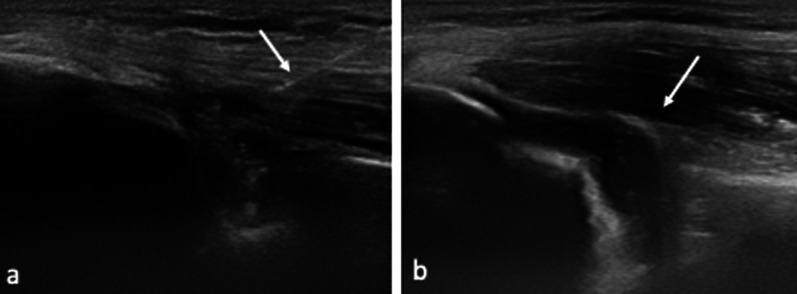
In-plane US-guided intra-articular injection (**a**). The spinal needle is clearly visible as a hyperechoic linear image, as the tip (white arrow) approaches the designed target. The posterior articular recess (asterisk) expands as contrast agent is injected, becoming visible as a hypoechoic sac, confirming the appropriateness of the procedure (**b**)

When intra-articular positioning of the needle is obtained, 5–7 mL of iodinated contrast agent (iopamidol 33 mg/mL), diluted to 60% with saline, is injected. Intra-articular needle tip positioning is confirmed by direct visualization (Fig. 3): injected fluid flows away from the tip with little resistance; if excessive resistance is encountered during injection and contrast does not flow into the posterior articular recess as expected, the needle should be slightly withdrawn and repositioned.

**Fig. 4:**
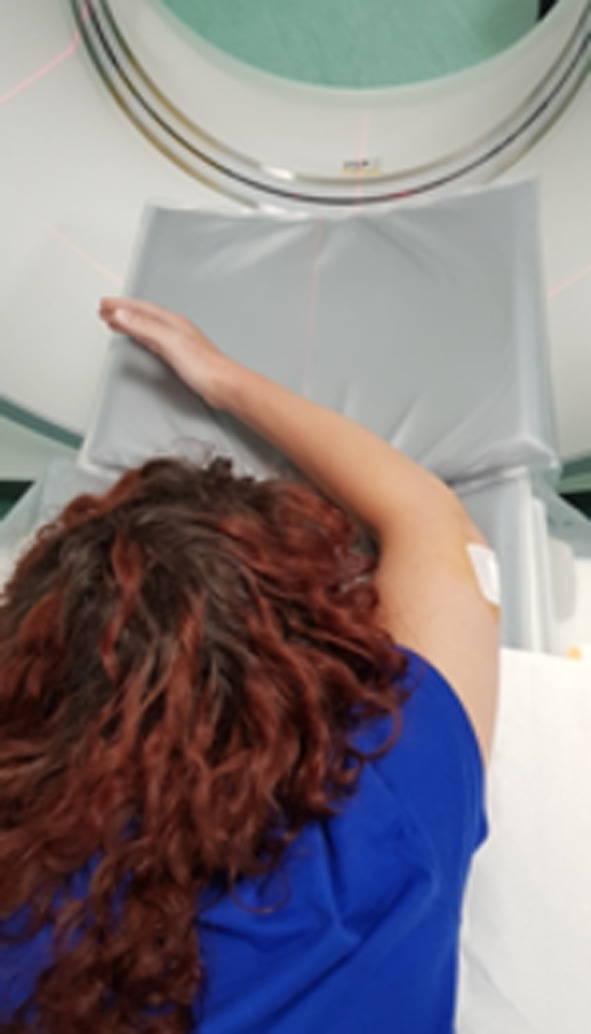
The patient is positioned prone on the CT scanning table, with the affected upper limb elevated over the head and with a 45° flexion of the elbow

Spiral CT is performed immediately after joint injection. We employed a 64-slice CT scanner (GE Revolution EVO). The patient is positioned prone on the CT scanning table, with the affected upper limb elevated over the head and with a 45° flexion of the elbow (Fig. 4). Flexion ensures ligament tension, improving their visualization on the CT arthrogram.

A 0.625 mm helical scan was obtained from the distal humerus, above the epicondyles, to the proximal radius and ulna, below the tuberosities (scan range 130 mm). The effective dose is approximately 0.2 mSv.

Axial images were then reformatted into coronal and sagittal planes, using a 1.25 mm slice-thickness and applying a bone window level. The isotropic data set can deliver multiplanar images with elevated spatial resolution and without spatial distortion artifacts, making this modality a superlative radiological tool in terms of anatomical detail.

The use of high-density iodinated contrast agent enhances the visualization of intra-articular structures, such as cartilage and ligaments, in relation to their low attenuation compared to the contrast agent, generating high-contrast imaging.

Rapidity is also one of the advantages of CT-arthrography, compared to MR-arthrography. A CT scan of the elbow requires just a few seconds of still posture, while MRI scanning requires the patient to be completely motionless for over half-an-hour, while in a narrow, frequently unpleasant gantry. This condition may be unbearable for certain patients with severe elbow pain or affected by claustrophobia.

Moreover, CT-arthrography can be adopted in patients with contraindications to MRI scanning, such as pacemakers, dorsal column stimulators, retained metal fragments, or implants classified as MRI-unsafe.

The overall timing of CT-arthrography combined to US-guided injection of contrast agent is about 15–20 min.

## Overuse-related instability of the elbow: ligament derangement

Ligamentous injuries of the elbow can be either caused by repetitive microtraumatic activities or by a single acute traumatic event, such as an elbow dislocation. We focused on injuries caused by repetitive activities that produce overuse damage: chronic overuse implies repetitive microtrauma of the elbow and can occur in both athletes and nonathletes.

Even though diagnosis is often made at clinical examination, imaging is helpful to confirm clinical interpretation, grade the injury, and guide treatment. The radiologist should be able to identify commonly seen patterns of injury since different structures are variably involved (tendons, ligaments, bones, cartilage).

In our article, we decided to focus specifically on ligamentous injuries secondary to overuse. In this setting, instability may affect the medial compartment, involving the MCL, or the lateral compartment, involving the LCL.

### Medial compartment: major elbow instability and posteromedial elbow impingement

Medial major elbow instability typically affects patients involved in athletic performances and results from acute or chronic injuries to the MCL. The most frequent presentation is chronic elbow pain localized to the medial side and valgus instability, worsened by overhead activities. Overhead throwing athletes, such as baseball pitchers, are particularly subjected to repetitive microtrauma to the MCL: the acceleration phase of throwing is characterized by great valgus and extension forces, leading to major tensile stress on medial structures, compressive forces on lateral structures, and shear forces posteriorly.

**Fig. 5:**
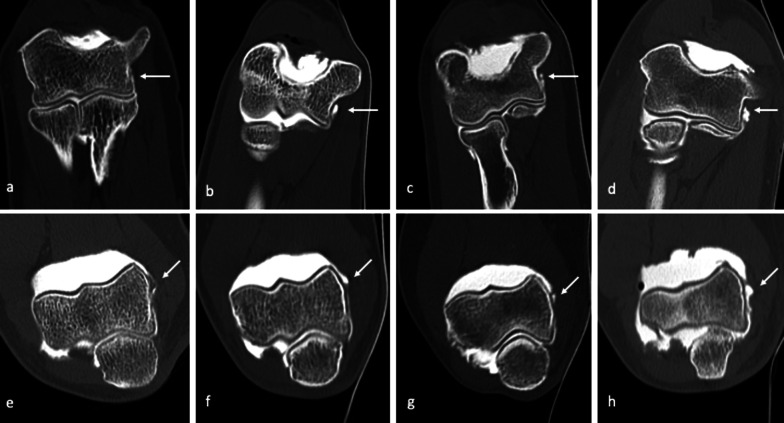
CT-arthrography coronal images (**a**–**d**) and axial images (**e**–**h**) belonging to four different patients, three of which display different aspects of ligament derangement involving the A-MCL (white arrows). Intact anterior band (A-MCL) of the medial collateral ligament (MCL) complex (**a**, **e** and **b**, **f**). Widening of the medial aspect of the articular recess, associated to a “bow-shaped” loose A-MCL, with no evidence of fiber tearing, suggestive of ligament laxity (**b**, **f**). Thickened and irregular A-MCL, showing a partial-thickness tear (**c**, **g**) and a full-thickness tear (**d**, **h**); contrast agent progressively spills through the ligament, as fibers are gradually disrupted as a consequence of micro-tearing and damage progresses from a partial to a complete tear

Surrounding bony structures and muscles acting as dynamic stabilizers reduce the stress distribution through the MCL by about half [43], but as these muscles fatigue, the amount of force transmitted to the MCL increases. The chronic tensile forces involved lead to inflammation, micro-tearing, and patholaxity of the ligament, which may eventually progress to disruption of the MCL (Fig. 5).

A combined valgus-extension overload can result in formation of posteromedial osteophytes that give rise to posterior elbow pain, as well as ulnar nerve irritation symptoms. Less commonly, the MCL may be injured after a traumatic elbow dislocation [44].

**Fig. 6:**
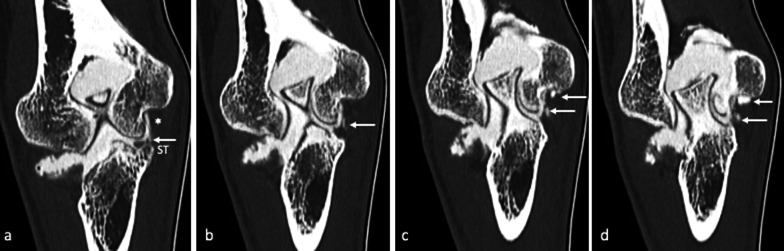
Professional tennis player with micro-tearing of the anterior band (A-MCL). Oblique coronal reformatted images depicting the A-MCL along its longitudinal axis, up to its insertion at the sublime tubercle (ST), highlight a thickened ligament (asterisk) with multiple strands of contrast enhancement within its fibers, due to micro-tearing (white arrows)

**Fig. 7:**
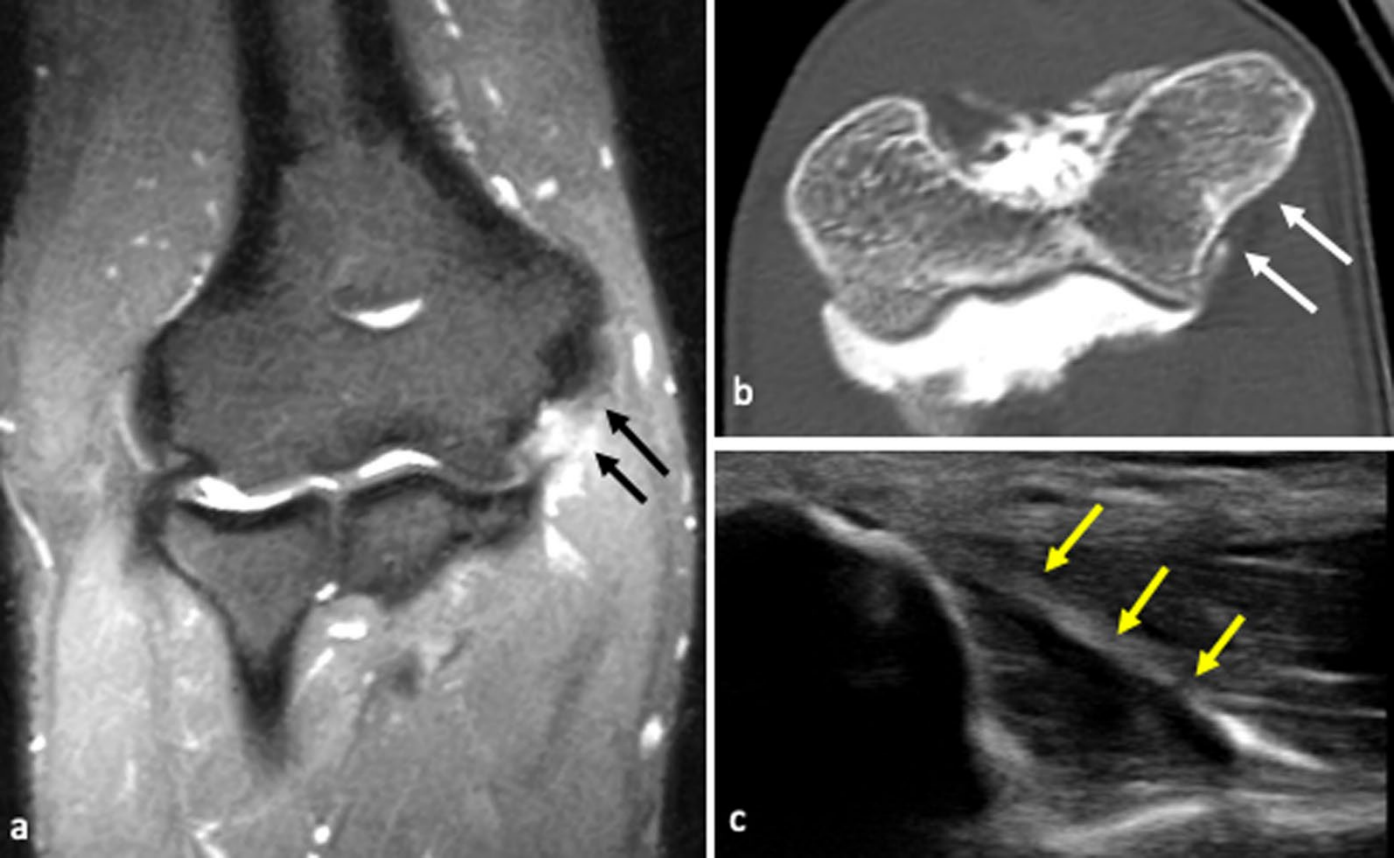
Professional tennis player with chronic medial elbow pain, exacerbated during serve, not responsive to medical and physical treatments. MRI coronal fat-suppressed T2-weighted images (**a**) show a high-intensity signal at the anterior bundle (A-MCL), suggestive of partial tear (black arrows). CT-arthrography reformatted images along the major axis of the anterior bundle (**b**) demonstrate a thickened but continuous ligament (white arrows). US scan prior to contrast agent injection (**c**) confirms diagnosis by showing a thickened, hypoechoic ligament with irregular margins, although regularly inserted (yellow arrows)

The anterior bundle of the MCL (A-MCL) is the main stabilizer against valgus stress; as such, it is most frequently injured in tennis players and baseball pitchers, due to the high forces involved in the tennis serve and the late-cocking phase of throwing, respectively (Figs. 6, 7) [4, 30, 33, 45].

MCL injuries are generally well-tolerated during daily activities. On the other hand, athletic performance can be severely impaired by either medial or posteromedial instability.

Posteromedial elbow impingement, also known as valgus extension overload (VEO) syndrome, is a rather uncommon disorder in the general population; however, it is a cause of disability in the overhead throwing athlete [46, 47].

**Fig. 8:**
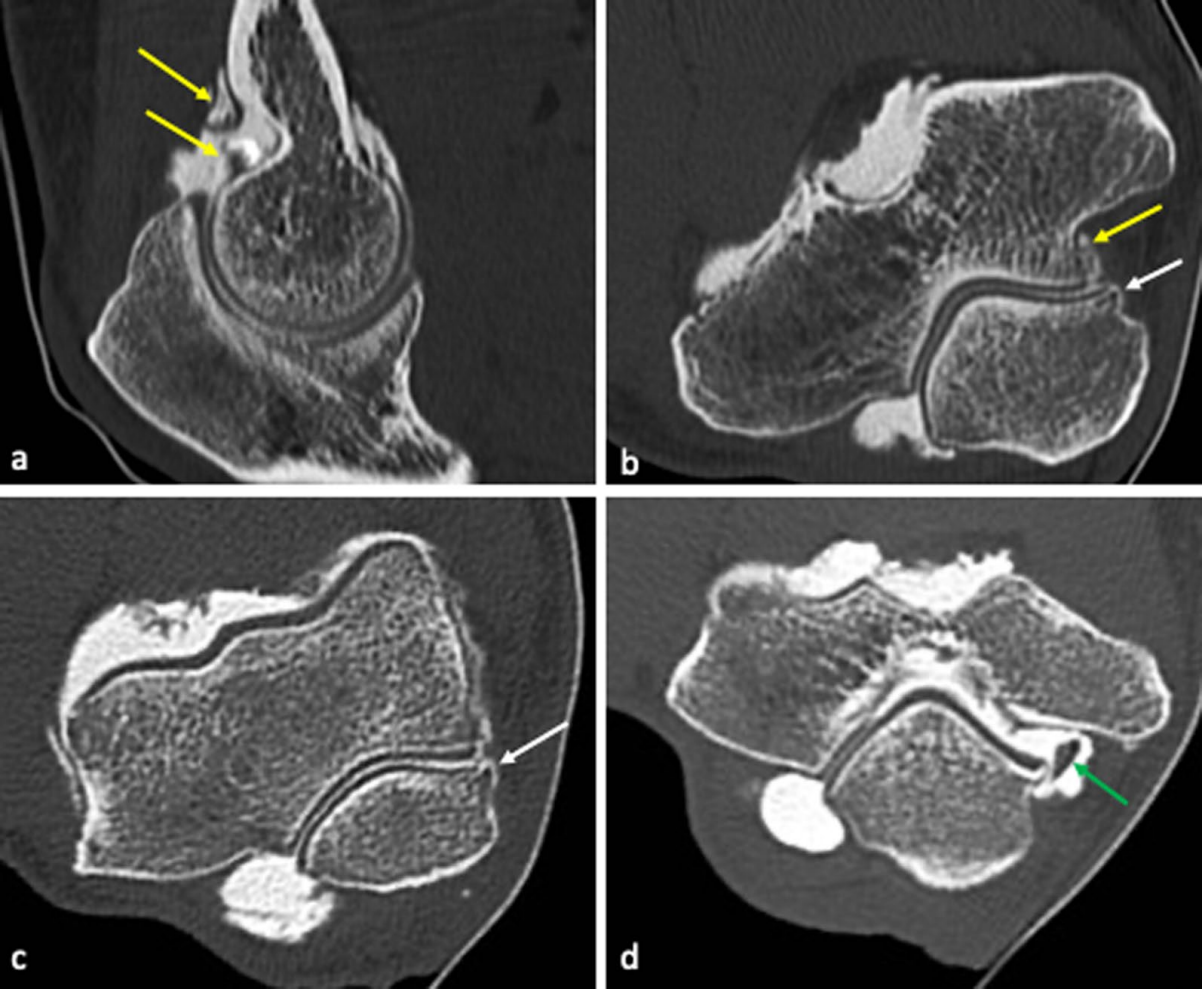
CT-arthrography sagittal (**a**) and axial (**b**–**d**) images of two different patients with valgus extension overload (VEO) syndrome, showing osteophyte formation (**b**, **c**) over the medial aspect of the olecranon (white arrows) and an intra-articular loose body (**d**) located medially at the posteromedial recess (green arrow). Synovial thickening associated to synovitis (**a**, **b**) is also noticeable (yellow arrows)

As previously mentioned, progressive medial laxity may occur from increased and repetitive combined hyperextension, valgus stress, and supination of the elbow. Such repetitive overload at the level of the posteromedial fossa may lead to posteromedial impingement, a bony and soft tissue mechanical abutment in the posterior fossa of the elbow, resulting in focal synovitis and olecranon spurring (Fig. 8a) [48].

The resultant soft tissue swelling, loose bodies, or osteophyte formation, or a combination of these, associated with abutment may result in symptoms localized to the posterior side of the elbow [46, 47, 49].

Over time, osteophytes may fracture, leading to loose bodies and mechanical symptoms (Fig. 8b).

The athlete complains of posterior pain, joint effusion, locking, crepitus, and a decrease in range of motion, most notably an extension deficit [49, 50]. Physical examination shows posteromedial tenderness and/or synovitis with possible associated extension loss and/or MCL laxity [51].

Posteromedial impingement can also be associated with ligament-related elbow instability, especially MCL insufficiency; it may also present in the setting of a rather stable MCL with a certain degree of developmental laxity [52].

### Lateral compartment: major (PLRI) and minor (SMILE) elbow instability

The LCL complex stabilizes the elbow against excessive varus and external rotational stress. Varus stress applied to the elbow is more common in the setting of an acute injury and only rarely related to a repetitive stress, which is more common at the medial compartment.

Tears can involve one or more of the three bundles, but the LUCL is the most important in terms of stability [53]. However, kinematic studies have described the LUCL and the RCL as working in concert to resist varus stress.

**Fig. 9:**
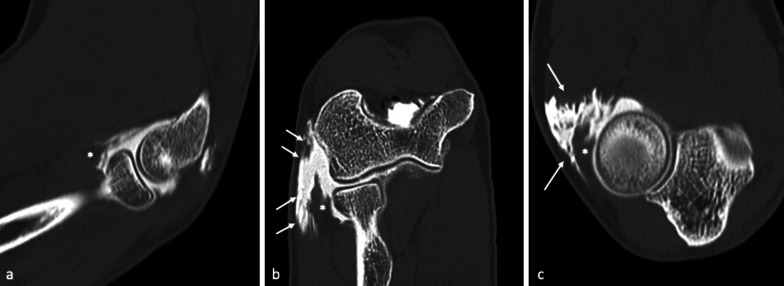
Posterolateral rotatory instability (PLRI) sagittal, coronal and axial images (**a**–**c**). Full-thickness tear of the lateral collateral ligament (LCL) complex (white arrows) with extra-articular leakage of iodinated contrast medium within fibers of the common extensor tendon (asterisks)

Damage to the LCL complex can lead to posterolateral rotatory instability (PLRI) of the elbow (Fig. 9), which is considered one of the major elbow instabilities involving the lateral compartment. This condition, as first described by O'Driscoll et al. [54], results in transient external rotatory subluxation of the ulna on the humerus, producing both humero-radial and humero-ulnar instability.

This represents the most common pattern of recurrent elbow instability, especially in the setting of chronic symptoms [55]; recurrent symptoms of lateral pain, locking, snapping, or popping may be present.

The feeling of instability most commonly occurs when the elbow is actively moved from flexion into extension, with a supinated forearm.

The primary cause of PLRI involves the partial or complete disruption of the LCL complex, which usually results in its avulsion off the lateral epicondyle [56] and is typically the result of trauma. A posterolateral luxation of the elbow can thus lead to chronic PLRI.

Other causes of injury to the LCL complex include chronic cubitus varus, sequelae of corticosteroid injections employed in the treatment of lateral epicondylitis, connective tissue disease [57–60], and/or other iatrogenic causes.

**Fig. 10:**
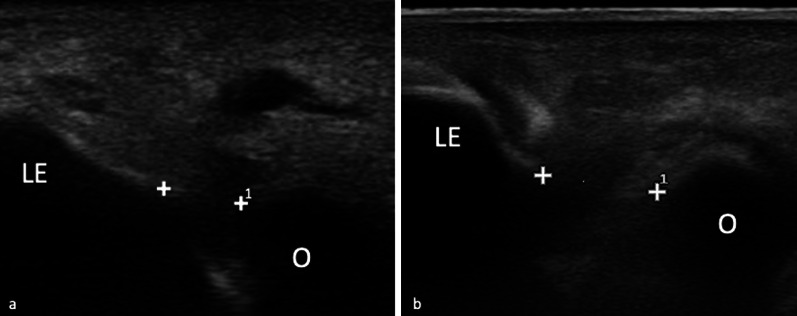
Posterolateral rotatory instability (PLRI) at dynamic US examination. The probe is placed in an axial approach between the lateral epicondyle (LE) and the olecranon (O), as the humero-ulnar joint is visualized. While applying a posterolateral rotatory stress, widening of the humero-ulnar joint is determined (white calipers): a widening greater than 4 mm, from resting (**a**) to stress (**b**) conditions, is considered indicative of PLRI

Dynamic ultrasound can be used to confirm clinical suspicion of instability (Fig. 10).

**Fig. 11:**
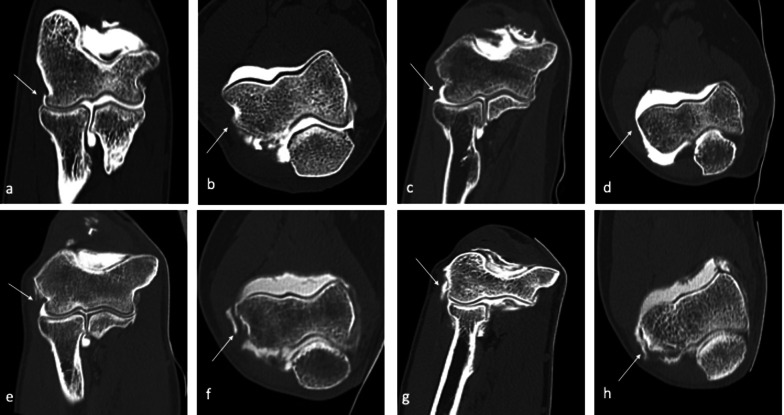
CT-arthrography coronal (**a**, **c**,** e**,** g**) and axial (**b**,** d**,** f**,** h**) images belonging to four patients, three of which display different aspects of symptomatic minor instability of the lateral elbow (SMILE), involving the RCL (white arrows). Intact radial band (RCL) of the lateral collateral ligament (LCL) complex (**a**, **b**). Widening of the lateral aspect of the articular recess associated to a curve-shaped, loose RCL with no evidence of fiber tearing, suggestive of patholaxity (**c**, **d**). Thickened and irregular RCL showing partial-thickness tear (**e**, **f**) and full-thickness tear (**g**, **h**); iodinated contrast progressively leaks through the ligament, as fibers are gradually disrupted and damage progresses from a partial to a full-thickness tear

**Fig. 12:**
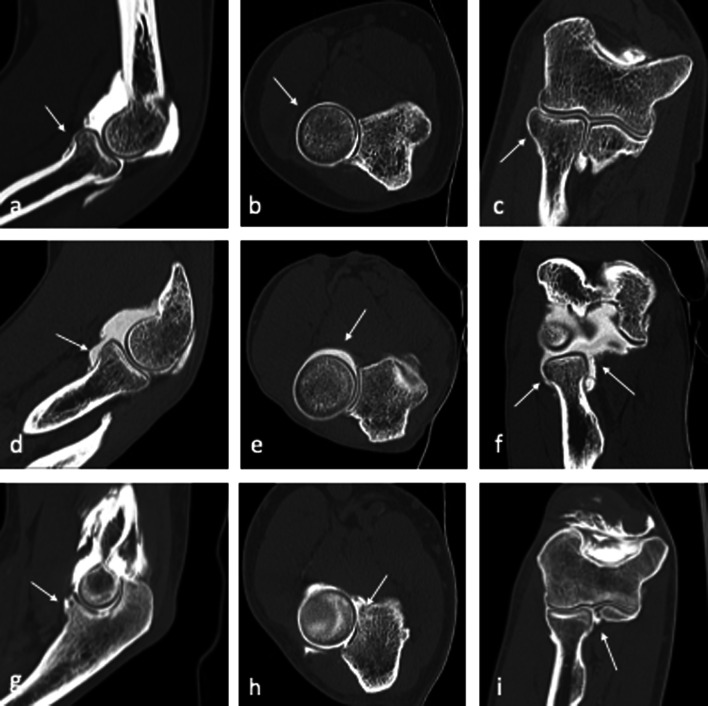
CT-arthrography sagittal (**a**, **d**,** g**), axial (**b**,** e**,** h**) and coronal (**c**,** f**, **i**) images belonging to three patients, two of which display different SMILE stages involving the AL (white arrows). Intact annular ligament (AL) of the lateral collateral ligament (LCL) complex (**a**–**c**). Loose AL with no evidence of fiber tearing, displaying the “loose collar sign”, suggestive of patholaxity (**d**–**f**). Thickened and irregular AL showing a partial-thickness tear (**g**–**i**); ligament fibers appear delaminated as iodinated contrast permeates through their layers

**Fig. 13:**
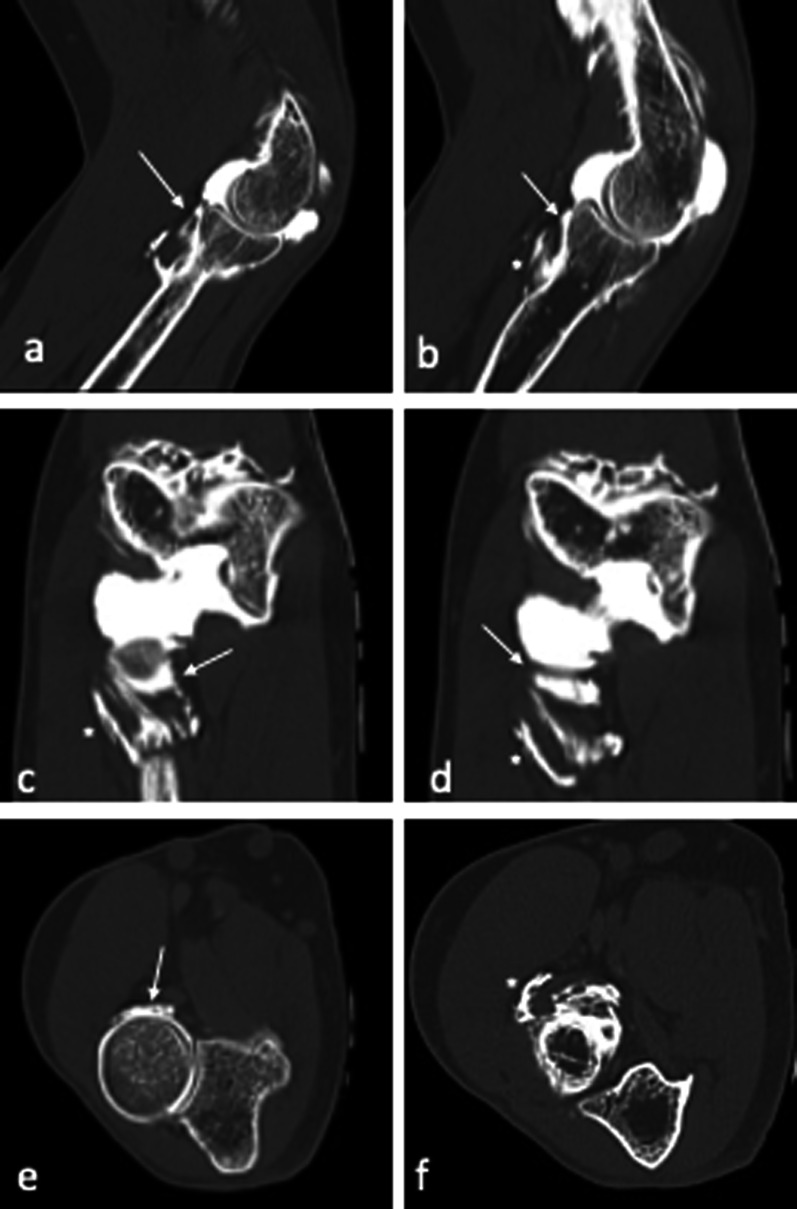
Symptomatic minor instability of the lateral elbow (SMILE) with a high-degree lesion of the annular ligament (AL) of the LCL complex. Consecutive sagittal (**a**, **b**), coronal (**c**, **d**) and axial (**e**, **f**) CT-arthrography scans highlight the ruptured AL (white arrows) and display extra-articular leakage (asterisks) through the damaged articular capsule

Recalcitrant lateral elbow pain, mostly diagnosed as lateral epicondylitis, is associated with a high incidence of intra-articular findings which may be related to a condition of patholaxity termed “symptomatic minor instability of the lateral elbow” (SMILE) (Figs. 11, 12, 13) [61].

This condition may result from repetitive low-energy stress or shear forces, occurring in simple, repetitive or prolonged daily or working activities performed with the shoulder in moderate abduction, pronation of the hand and 50°–70° of elbow flexion. In this position, the hand and the forearm create a varus/pronation load on the lateral elbow, with progressive stretching and elongation of the RCL and the annular ligament, both associated to a relative hypermobility of the radial head.

## Conclusions

Even though plain MRI and MR-arthrography remain the reference standard for the evaluation of overuse-related instability of the elbow, CT-arthrography represents a solid alternative whenever MRI is unavailable or clinically unfeasible. Proper knowledge of CT-arthrography findings is thus essential in order to accurately interpret pathological findings.

In these instances, CT-arthrography and pre-procedural US examination represent valuable and complementary diagnostic tools, especially when supplemented by dynamic US evaluation of ligaments through specific stress testing.

## Data Availability

Not applicable.

## Abbreviations

AL: Annular ligament
A-MCL: Anterior bundle of the medial collateral ligament
CT: Computed tomography
LCL: Lateral (radial) collateral ligament
LE: Lateral epicondyle
LUCL: Lateral ulnar collateral ligament
MCL: Medial (ulnar) collateral ligament
MRI: Magnetic resonance imaging
PLRI: Postero-lateral rotatory instability of the elbow
P-MCL: Posterior bundle of the medial collateral ligament
RCL: Radial collateral ligament
SMILE: Symptomatic minor instability of the lateral elbow
ST: Sublime tubercle
US: Ultrasound
VEO: Valgus extension overload
