# Correction: Overuse-related instability of the elbow: the role of CT-arthrography

**DOI:** 10.1186/s13244-022-01185-9

**Published:** 2022-03-10

**Authors:** Andrea Zagarella, Giulia Signorelli, Giulia Muscogiuri, Roberta Colombo, Gianluca Folco, Paolo Arrigoni, Mattia Radici, Pietro Simone Randelli, Mauro Battista Gallazzi

**Affiliations:** 1 U.O.C. Radiodiagnostica, Azienda Socio Sanitaria Territoriale Centro Specialistico Ortopedico Traumatologico Gaetano Pini-CTO, Piazza Cardinal Ferrari 1, 20122 Milan, Italy; 2 Scuola Di Specializzazione in Radiodiagnostica, Università Degli Studi Di Milano, Via Festa del Perdono 7, 20122 Milan, Italy; 3 I Clinica Ortopedica, Azienda Socio Sanitaria Territoriale Centro Specialistico Ortopedico Traumatologico Gaetano Pini-CTO, Piazza Cardinal Ferrari 1, 20122 Milan, Italy; 4 Scuola Di Specializzazione in Ortopedia e Traumatologia, Università Degli Studi Di Milano, Via Festa del Perdono 7, 20122 Milan, Italy; 5 Laboratory of Applied Biomechanics, Department of Biomedical Sciences for Health, Università Degli Studi Di Milano, Via Mangiagalli 31, 20133 Milan, Italy; 6 U.O.C. 1° Clinica Ortopedica, ASST Centro Specialistico Ortopedico Traumatologico Gaetano Pini-CTO, Piazza Cardinal Ferrari 1, 20122 Milan, Italy; 7 Research Center for Adult and Pediatric Rheumatic Diseases (RECAP‑RD), Department of Biomedical Sciences for Health, Università Degli Studi Di Milano, Via Mangiagalli 31, 20133 Milan, Italy

## Correction to: Insights Imaging (2021) 12:140 https://doi.org/10.1186/s13244-021-01065-8

The original article contained a numerical error in the Technique sub-section. The value ‘0.625 mm’ was corrected from ‘6.25 mm’. https://doi.org/10.1186/s13244-021-01065-8

